# Acute respiratory distress syndrome in patients with COVID-19 vs. Non-COVID-19: clinical characteristics and outcomes in a tertiary care setting in Mexico City

**DOI:** 10.1186/s12890-023-02744-6

**Published:** 2023-11-06

**Authors:** Paul Palacios-Moguel, Alejandra Esquivel-Pineda, Xavier A. Flores-Andrade, Janet S. Aguirre-Sanchez, Nancy N. Cruz-Arellanes, Julio C. Sauza-Sosa, Naybeth García-Gonzalez, Daniel Manzur-Sandoval, Enma Toledo-Aleman, Edgar García-Cruz

**Affiliations:** 1 Critical Care Unit, ABC Medical Center, Mexico City, Mexico; 2 Centre for Research in Infectious Diseases (CIENI) of the National Institute of Respiratory Diseases Ismael Cosío Villegas (INER), Mexico City, Mexico; 3 Cardiology Department, ABC Medical Center, Mexico City, Mexico; 4 Cardiovascular Critical Care Unit, National Institute of Cardiology Ignacio Chávez, Mexico City, Mexico

**Keywords:** COVID-19, ARDS, ICU, Mortality, Right ventricular dysfunction

## Abstract

**Background:**

Acute Respiratory Distress Syndrome (ARDS) due tocoronavirus disease (COVID-19) infection has a unique phenotype generating a growing need to determine the existing differences that can alter existing evidence-based management strategies for ARDS. Research Question: What differences does the clinical profile of patients with ARDS due to COVID 19 and Non-COVID 19 have?

**Study Design and methods:**

We conducted a comparative, observational, retrospective study in the Intensive Care Unit (ICU)of a third-level hospital in Mexico City, from March 2020 through March 2022. Clinical, echocardiographic, and laboratory variables were compared between patients with ARDS due to Severe Acute Respiratory Syndrome Coronavirus 2 (SARS-CoV-2) infection and those due to other etiologies.

**Results:**

We enrolled 140 patients with a diagnosis of ARDS. The study group of COVID-19 etiology were younger males, higher body mass index, progressed to organ dysfunction, required more frequently renal replacement therapy, and higher SOFA score. There was no difference in rates of right ventricular dysfunction.

**Interpretation:**

COVID-19 ARDS exhibit much greater severity that led to higher admission and mortality rates, whilst being younger and less comorbid.

**Supplementary Information:**

The online version contains supplementary material available at 10.1186/s12890-023-02744-6.

## Background

In 2019 the world experienced an unprecedented epidemiological event aroused by a novel coronavirus, causing millions of deaths due to Coronavirus Disease (COVID-19) disease. Physicians all around the world strived to treat patients with a disease caused by an unknown pathogen, while learning about the unique features of the disease, and causing immeasurable losses with great social impact, forcing the world to stop its everyday routine [1, 2].

This global threat encompasses a broad clinical picture characterized mainly by fever, cough, and dyspnea, in addition to a specific diagnostic test [3]. However, some cases can develop a severe disease, expressed by respiratory failure, rapidly progressive cytokine storm leading to widespread tissue damage and acute respiratory distress syndrome (ARDS) aggravation resulting in multi-organ failure and death [4]. Reports estimate that approximately 5% of COVID-19 patients require intensive care unit (ICU) management, which are associated with mortality rates 68% or higher [5, 6]. A pre-pandemic study exposed pneumonia as the underlying risk factor for non-COVID-19 ARDS, accounting for 59% of the cases. Recently, a retrospective study conducted in Wuhan, China, found that 41.8% of adult patients admitted to an ICU with COVID-19 pneumonia developed ARDS [6, 7].

Numerous studies have enlisted risk factors for increased mortality from COVID-19 pneumonia which include advanced age, ischemic heart disease, diabetes mellitus, and chronic kidney disease. In a collective effort to determine the severity with which patients present in the emergency room, attempts have been made to establish risk scores based on these risk factors and clinical presentation, to predict outcomes and anticipate treatment. Unfortunately, due to the rapid deterioration, no risk score has been successful in this task. However, lymphopenia, elevated ferritin, interleukin 6, and elevated acute phase proteins are predictors of a poor prognosis of the disease [8–10].

Postmortem lung histopathological analysis of patients with COVID-19 pneumonia have shown diffuse alveolar damage, which has previously been used to identify a specific ARDS phenotype with higher mortality [11]. Clearly, COVID-19 ARDS exhibit similarities to classic Non-COVID-19 ARDS. However, it is COVID-19 rapid progression, multisystem involvement, normal or increased ventilatory compliance, which lead to fatal outcomes [11–13].

There is paucity of data whether ARDS due to COVID-19 infection has a unique phenotype generating a growing need to determine if non-COVID-19 ARDS have differences with COVID-19 ARDS that can alter existing evidence-based management strategies for ARDS. The objective of the present study was to compare the characteristics and clinical outcomes of ARDS in COVID-19 and Non-COVID-19 patients.

## Methods

We conducted a comparative, observational, retrospective study in the ICU of a tertiary care setting in Mexico City from March 2020 through March 2022. Medical records of patients over 18 years old that met the diagnostic Berlin Definition for ARDS (PaO2/Fio2 ratio < 300 under positive end-expiratory pressure (PEEP)/continuous positive airway pressure (CPAP) > 5 cmH2O; acute onset within a week; bilateral shadows in the lung field and respiratory failure that cannot be explained by cardiac failure or excess fluid alone) and had complete echocardiographic and laboratory assessment were enrolled in the study and divided into two groups: COVID-19 positive patients confirmed by polymerase chain reaction (PCR) presenting with ARDS, and a non-COVID-19 ARDS presentation. The necessity of vasoactive amines, the presence of clinical signs of hypoperfusion, or requirement of mechanical ventilation were used as indicators for admission to the ICU. Patients with pre-existing chronic respiratory failure due to neuromuscular or neurologic disease, the presence of a tracheostomy, and incomplete medical records were considered exclusion criteria. It is important to mention that at the time of designing the present study, the total number of patients with ARDS was not known, especially patients with non-COVID-19 ARDS. Additionally, not all reports of echocardiograms performed were available for review. For this reason, it was decided to carry out convenience sampling.

Basal demographic and clinical characteristics, admission and discharge dates, anthropometric measurements, length of hospital and ICU stay, days on mechanical ventilation (MV), renal replacement therapy, and extracorporeal membrane oxygenation (ECMO) were collected from medical records. Biochemical tests (leukocytes, lymphocytes, D-dimer, C-reactive protein, procalcitonin, platelets, brain natriuretic peptide, and ultra-sensitive troponin levels) and transthoracic echocardiogram (basal, mean, and longitudinal right ventricular (RV) diameter, tricuspid annular plane systolic displacement (TAPSE), s’, pulmonary artery systolic pressure (PASP), TAPSE/PSAP, left ventricular ejection fraction, E/a, E/e’, and right ventricular shortening fraction), performed in a window of 24 h post-intubation, were collected from the medical records. Similarly, and within the same 24-hour window, the following scales were calculated: SAPS-II, APACHE-II, and SOFA. Data was collected and stored in a Microsoft Excel database. Only patients with complete medical records were included.

A statistical analysis was performed using the SPSS 25.0 statistical package. The qualitative variables are presented with frequencies and percentages. For the quantitative variables, the Kolmogorov-Smirnov normality test was used to determine distribution normality. For variables with a normal distribution, mean and standard deviation were used to represent the data; for the variables that followed a non-normal distribution, median and ranges were used. Clinical, echocardiographic, and laboratory variables were compared between patients with ARDS due to SARS-CoV-2 infection and those due to other etiologies. For qualitative variables, the chi-square test was used. For quantitative variables with a normal distribution, a t-test was performed, while for those with a non-normal distribution, a Mann-Whitney U test was used, considering a level of significance of 0.05. A univariate and multivariate logistic regression model was proposed to analyze the outcomes in relation to the patients’ comorbidities.

ECMO and renal replacement therapy data were included for analysis. In addition, data on right heart failure (rapidly progressive syndrome characterized by systemic congestion resulting from impaired right ventricular filling and/or reduced right ventricular emptying) was considered. In our study, prolonged MV was defined as unsuccessful extubating after three or more spontaneous breathing tests or more than seven days of MV, according to the WIND study [14].

The objective of the present study was to compare the characteristics and clinical outcomes of ARDS in COVID-19 and Non-COVID-19 patients. This study was submitted for review by the research and ethics committees of the medical center, obtaining approval for its development.

## Results

One hundred and forty patients with ARDS enrolled in the study, of which 79 (56%) had a diagnosis of COVID-19 infection. Male patients constituted 68.6% (96) of the study sample and 31.4% (44) were female, presenting a mean age of 67.5 years (IQR 25–97). Body mass index was calculated for every participant, having a mean 27.34 kg/m2 (IQR 13–43). The risk scores of both groups were a median of 33 for SAPS-II, APACHE II of 14 points and SOFA of 7 points. A total of 100 patients (71.4%) required MV, with a median duration of 6 days (IQR 0–64). A general mortality of 32.1% (45 patients) was documented. Table 1 shows clinical characteristics of the patients included and comorbidities. Regarding the echocardiographic parameters, only the basal diameter of the right ventricle (median 41 mm) and pulmonary artery systolic pressure (median 40mmHg) revealed abnormalities, with the rest of the echocardiographic values ​​within normality (Table 2).

**Table 1:** General characteristics of the patients in the study

Characteristic	Total (140)	Non-COVID-19 (n = 61)	COVID-19 (n = 79)	*p*
Sex ^a^ Male Female	96 (68.6) 44 (31.4)	34 (55.7) 27 (44.3)	62 (78.5) 17 (21.5)	< 0.01
Age ^b^	67.5 (25–97)	72 (25–97)	64 (32–89)	0.03
Weight (kg)	76 (36–130)	70 (36–130)	80 (44–127)	< 0.01
BMI (kg/m^2^) ^b^	26.34 (13–43)	24.56 (13–43)	27.34 (18–43)	˂0.01
Comorbidities ^a^ Diabetes Systemic Hypertension COPD Heart Failure Ischemic Heart Disease	31 (22.1) 71 (50.7) 10 (7.1) 7 (5) 12 (8.6)	8 (13.1) 28 (45.9) 8 (13.1) 4 (6.6) 5 (8.2)	23 (29.1) 43 (54.4) 2 (2.5) 3 (3.8) 7 (8.9)	0.02 0.31 0.01 0.45 0.88
SAPS-II score ^b^	33 (6–89)	32 (6–70)	36 (10–89)	0.07
APACHE-II score ^b^	14 (0–38)	13 (0–30)	14 (5–38)	0.68
SOFA score ^b^	7 (0–90)	5 (0–9)	8 (2–18)	0.01
PaO2/FiO2	163 (133–226)	185 (135–229)	158 (109–221)	0.10
ARDS classification ^a^ Mild Moderate Severe	52 (37.1) 71 (50.7) 17 (12.1)	25 (40.9) 33 (54) 3 (4.9)	27 (34.1) 38 (48.1) 14 (17.7)	0.06
RV failure ^a^	9 (6.4)	4 (6.6)	5 (6.3)	0.95
Mechanical Ventilation ^a^	100 (71.4)	22 (36.1)	78 (98.7)	˂0.01
Renal Replacement Therapy ^a^	16 (11.4)	3 (4.9)	13 (16.5)	0.02
ECMO ^a^	1 (0.7)	0	1 (1.3)	0.37
Duration Mechanical Ventilation ^b^	6 (0–64)	0.88 (0–18)	13 (0–64)	˂0.01
Days in Critical Care Unit ^b^	9.5 (0–68)	2 (0–30)	17 (1–68)	˂0.01
In-Hospital stay ^b^	13.5 (1–68)	6 (1–31)	22 (1–68)	˂0.01
Deaths ^a^	45 (32.1)	12 (19.7)	33 (41.8)	˂0.01

Data presented as: ^a^ total of patients (%), ^b^ median (interquartile range). Abbreviations: BMI: body mass index, SAPS-II: Simplified Acute Physiology Score II, APACHE-II: Acute Physiology and Chronic Health Evaluation II score, SOFA: Sequential Organ Assessment score, COPD: Chronic Obstructive pulmonary disease, RV: right ventricular, ECMO: Extracorporeal membrane oxygenation. Statistical analysis: U Mann-Whitney test was performed for numeric variables, and Chi square test was used for nominal/ordinal variables

**Table 2:** General echocardiographic characteristics

Echocardiographic characteristics	Total	Non-COVID-19 (n = 61)	COVID-19 (n = 79)	*p*
RV basal diameter (mm)	41 (23–65)	42 (32–57)	40 (23–65)	0.36
RV average diameter (mm)	33 (18–60)	34 (20–47)	32.81 (18–60)	0.02
RV longitudinal diameter (mm)	76 (58–96)	77 (60–94)	76 (58–96)	0.32
TAPSE	22 (9–34)	21 (10–34)	22 (9–33)	0.36
RV S wave	12.8 (5.3–24)	12.7 (7-17.8)	13 (5.3–24)	0.52
PSAP	40 (20–97)	42 (20–82)	40 (23–97)	0.23
Ventricular-arterial Uncoupling	0.53 (0.16–1.12)	0.52 (0.18-1)	0.55 (0.165–1.12)	0.25
RV ejection fraction (%)	65 (25–78)	59 (25–78)	65 (40–78)	˂0.01
E/A ratio	1 (0.3-3)	0.97 (0.3-3)	1 (0.46–2.44)	0.62
E/e’ ratio	9 (4.7–26)	10 (4.7–26)	8.7 (5-23.5)	0.09
RV FAC (%)	45 (6–70)	42 (6–57)	46.5 (20–70)	< 0.01

Data presented as: median (interquartile range) Abbreviations: RV: right ventricle, TAPSE: tricuspid annular plane systolic excursion; S wave: peak systolic tissue velocity at the tricuspid annulus, PASP: pulmonary artery systolic pressure, FAC: fractional area change. Normal values: RV basal diameter: ≤41 mm, RV average diameter < 35 mm, TAPSE: ≥17 mm, RV S wave: ≥9.5 cm/seg, E/e ratio: <14, E/A ratio: >2, PASP: <35 mm Hg. Statistical analysis: U Mann-Whitney test

The patient characteristics by group (shown also in Table 1) showed interesting differences. The ARDS due to COVID-19 group had younger patients (mean age 64 vs. 72). Higher body mass index was observed in the COVID-19 ARDS group (27.34 kg/m2 vs. 24.56 kg/m2). Regarding comorbidities, only diabetes mellitus (DM) and chronic obstructive pulmonary disease (COPD) showed statistical significance between the COVID-19 ARDS group and the Non-Covid-19 ARDS group. A greater number of patients in the COVID-19 ARDS group progressed to organ dysfunction expressed with a higher SOFA score (8 [IQR 2–18] vs. 5 [IQR 0–9]). The PaO2/FiO2 index did not show significant differences, however, it was higher in the group of non-COVID-19 ARDS patients (185[IQR 135–229] vs. 158 [IQR 109–221]). Patients were classified according to their PaO2/FiO2 as mild, moderate and severe according to the Horowitz index classification, without showing a statistically significant difference between both groups.

There was a significant number of patients in the COVID-19 ARDS group requiring MV compared to the patients in the Non-COVID-19 ARDS group (78 [98.7%] vs. 22 [36.1%]). Furthermore COVID-19 patients required a greater number of days of MV (13 days [IQR 0–64] vs. 0.88 days [IQR 0–18]). In the COVID-19 ARDS group 13 (16%) patients required renal replacement therapy, significantly different to the Non-COVID-19 group with only 3 (5%). The total length of in-hospital stay (22 [IQR 1–68] vs. 6 [IQR 1–31]) and at the ICU (17 [IQR 1–68] vs. 2 [IQR 0–30]) was greater in the COVID-19 ARDS group. Mortality was higher in COVID-19 ARDS group, accounting for 33 (41%) compared with 12 (20%) in the Non-COVID-19 ARDS group.

There was no difference in the laboratory findings between the two groups, except leukocytes, procalcitonin and troponin. Leukocyte count was reported higher in COVID-19 ARDS patients with a median of 11.1 (IQR 1.9–34) compared to 8.5 (IQR 0.1–23.3) of the Non-COVID-19 patients. Patients in the Non-COVID-19 group, presented higher levels of procalcitonin (0.54 [IQR 0.02–100] vs. 0.26 [IQR 0.002-8.3]) and troponin (30.5 [IQR 2.4–903] vs. 16.7 [IQR 3-111]). The rest of the laboratory work did not show statistical differences (Table 3).

**Table 3:** Laboratory findings

Characteristic	Total (140)	Non-COVID-19 (n = 61)	COVID-19 (n = 79)	*p*
Leukocytes (x10^9^ /L)	10.15 (0.1–34.0)	8.5 (0.1–23.3)	11.1 (1.9–34)	˂0.01
Lymphocytes (x10^9^ /L)	0.7 (0.0-11.8)	0.7 (0-2.8)	0.6 (0-11.8)	0.69
D-dimer (ng/mL)	1837 (150-31820)	2303 (150–4320)	2947 (311-31820)	0.22
C- reactive protein (mg/L)	12.82 (0.1–54.2)	13.5 (0.1–42)	12.4 (0.1–54.2)	0.65
Procalcitonine (ng/mL)	0.3 (0.02–100)	0.54 (0.02–100)	0.26 (0.02–8.3)	< 0.01
Platelets (x10^3^/L^)^	213 (21–970)	200 (26–970)	223 (21–548)	0.52
NT-proBNP (pg/ml)	1032 (16-35000)	1291 (58-35000)	714 (16-30370)	0.17
Troponin I (pg/ml)	25 (2.4–1119)	30.5 (2.4–903)	16.7 (3-1119)	˂0.01

Data presented as: median (interquartile range) Abbreviations: NT-Pro BNP: N-terminal B-type natriuretic peptide. Statistical analysis: U Mann-Whitney test

For the echocardiographic analysis, only the left ventricle ejection fraction (59 [IQR 25–78] vs. 65 [IQR 40–78]) and the right ventricular fractional area change (42 [IQR 6–57] vs. 8.7 [IQR 5-23.5]) presented statistical differences between groups. The rest of the echocardiographic parameters were no different between the COVID-19 ARDS and the Non-COVID-19 ARDS groups (shown also in Table 2).

As mentioned, 12 patients in the Non-COVID-19 ARDS group and 33 patients in the COVID-19 ARDS group died. From this subset of patients, statistical differences were observed in requirements of MV (p < 0.01), renal replacement therapy (p 0.04), duration of MV (p < 0.01), ICU days (p < 0.01) and in-hospital stay (p < 0.01), all being greater in the COVID-19 ARDS group as shown in Table 4. However, in the univariate and multivariate logistic regression model, comorbidities showed no statistical significance as a risk factor for mortality in both groups. (Results are depicted in section “A”).

**Table 4:** Outcomes associated with mortality

Outcomes	Non-COVID-19 (n = 12)	COVID-19 (n = 33)	p
Comorbidities			
● Diabetes ● Systemic Hypertension ● COPD ● Heart Failure ● Ischemic Heart Disease	1 (8.3) 6 (50) 0 1 (8.3) 0	11 (33.3) 18 (54.5) 1 (3) 3 (9.1) 5 (15.2)	0.09 0.78 0.54 0.93 0.15
Mechanical ventilation ^a^	9 (75)	33 (100)	< 0.01
Renal replacement therapy ^a^	1 (8.3)	13 (39.3)	0.04
RV failure ^a^	2 (16.7)	2 (6.1)	0.26
Duration of mechanical ventilation ^b^	1 (43.7)	22 (52)	< 0.01
Days in Critical Care Unit ^b^	1 (28.1)	28 (47.9)	< 0.01
In-Hospital stay ^b^	6 (31.8)	29 (46)	< 0.01

Data presented as: ^a^ total of patients (%), ^b^ mean (standard deviation). Abbreviations: RV: right ventricular. Statistical analysis: U Mann-Whitney test was performed for numeric variables, and Chi square test was used for nominal/ordinal variables

## Discussion

As stated, we herein present a comparison between homogenous groups in which the mortality was fewer than the reported worldwide [15–19]. In Mexico, mortality rates were reported by Fernández et al. as high as 43% [20]. However, in a review by Salinas et al., a general mortality of 4.94% per 1000 person-years was reported [21]. The ICU mortality in Mexico increases considerably and has been reported up to 60%. In our cohort mortality was considerably lower than that presented by various study groups internationally. Something that significantly contributes as a viable explanation to this discrepancy is that all patients presenting with severe COVID-19 infection to the emergency department were admitted to a COVID-19 dedicated ICU, without delay. This was a compelling factor due to the concurrent evaluation on mechanical ventilation abilities and knowledge of the medical and non-medical staff working at the ICU.

We emphasize that at the beginning of the study, vaccines and treatments, such as IL-6 inhibitors and some antivirals, were not available in Mexico. However, by mid 2021, the use of vaccines was according to age groups and comorbidities, which meant that not the entire population was vaccinated. Similarly to the vaccines, the use of IL-6 inhibitors and antivirals was not universal since there was no absolute recommendation on their effectiveness.

In many studies, it has been reported that patients who presented themselves with Reduced RV Systolic Function and a diminished Ventricular-Arterial Coupling associated with ARDS COVID-19 (TAPSE/PSAP26, 27) had a worse morbidity/mortality prognosis, however within our investigation, these results were not confirmed.

Regarding the basal biochemical and laboratory profile, differences were only observed in leukocytes, procalcitonin, and troponin I. The two latter biochemical parameters were higher in Non-COVID-19 ARDS patients, contrary to what was reported by various authors who mention that COVID-19 infection produces elevation of troponin I by various mechanisms and is associated in a high percentage with RV dysfunction [22]. We hypothesize that this discrepancy is due to the fact that our ICU receives patients with a great myriad of affections, being the most frequent causes of admission: sepsis, oncological diseases and cardiovascular diseases, which are pathologies that are frequently associated with cardiovascular dysfunction and consequently elevation of biochemical markers (troponins, D-dimer, NT-pro BNP).

When comparing outcomes in both groups, COVID-19 vs. Non-COVID-19 ARDS, statistical significance was observed in mechanical ventilation requirements and its duration. Data that correlates with other publications, confirming that COVID-19 ARDS exhibit much greater severity that led to higher admission rates, whilst being younger and less comorbid. As reported by Bain et al. in a cohort of 27 patients with Covid-19 ARDS, where it was found that patients with Covid-19 ARDS were released from mechanical ventilation later, the mean being 13.5 (8.0–18.0) for Covid-19 and 8.0 (5.0–25.0) for non-covid-19 ARDS, P 0.06 [22]. When analyzing the comorbidities by group of patients, no significant differences were observed as risk factors that could increase mortality, this suggests that the severity is greater in ARDS COVID 19 compared to Non-COVID19 ARDS regardless of the associated comorbidities in the patients.

This study also coincides with previous data relating with the rates of multisystemic involvement (shown by the SOFA score), which is rapidly progressive. We highlight that, even almost four years later, this data should be analyzed taking into account the multiple hypotheses generated for this phenomenon.

Contrary to the existing data, our rates of renal replacement therapy (16.5%) are lower in the COVID-19 ARDS group [22, 23]. It is important to mention that the severity and evolution of the disease depend on the comorbidities of each patient and the available equipment in the care setting. García et al. reported in a cohort study, that 34% of patients developed acute kidney injury and 12% required renal replacement therapy [24].

Right ventricular dysfunction (RVD) is common in patients with ARDS as a consequence of pulmonary hypertension [25]. Transthoracic echocardiography is a useful tool for the timely diagnosis and early treatment of patients with this condition. It is currently well known that patients with RVD associated with ARDS have a worse prognosis [26–28]. In our study, no association of RVD between groups was found, contrary to what has been reported in the literature.

A simple regression model was conducted in which the main possible comorbidities were analyzed in both groups (DM, HTN, COPD, HF, CAD). These comorbidities did not increase the risk of morbidity/mortality. When using a multivariate analysis, no statistical significance was observed when correlating these comorbidities with an increased risk of complications, within both groups.

In 2021 Bain et al. compared the main clinical and biochemical characteristics, and outcomes in a cohort of patients diagnosed with COVID-19 and non-COVID-19, finding a greater number of days of mechanical ventilation in the patients with COVID-19, with no evidence of significant difference in mortality [1].

No scientific evidence was obtained to modify traditional ARDS management in these patients, however in this study a higher mortality in ARDS due to COVID-19 (41.8%) compared to non-COVID-19 ARDS (19.7%) is documented. The variables associated with higher mortality risk were the need for MV, renal replacement therapy, days on MV, ICU days, and days of hospitalization.

As we mentioned, global mortality was less than reported worldwide. We identify key points to this success: early instauration of mechanical ventilation helped to avoid injury due to P-SILI (pulmonary self-induced lung injury), use of the prone position in awake patients and non-invasive mechanical ventilation devices, strict adherence to alveolar protection measures preventing ergotrauma and increased right ventricular afterload.

Finally, it is important to mention that although there was no formal sample calculation, opting for convenience sampling, the statistical power of the study was calculated using the proportion of deaths in each group, obtaining a power of 79.93%.

### Strengths and limitations of the study

The retrospective nature of our study precluded the possibility of ascertaining causal relationships but did provide some rationale to raise questions about the prognosis of adult patients with COVID-19 ARDS. Furthermore, our research was conducted at a single center. It is necessary to point out that there is a limitation related to the patients admitted in the center’s ICU. Many of these patients come from the oncology center and the coronary unit which present a wide range of conditions to the ICU. Especially being important with the participants in the Non-COVID-19 group. This explains some of the data obtained, and we present it as a limitation to the study. As discussed above, although there was no formal sample calculation, the power of the study is considerable.

## Conclusions

ARDS is a disease with high mortality, irrespective of etiology. COVID-19 showed higher morbidity and mortality compared to the non-COVID-19 etiology. The need for mechanical ventilation, days on mechanical ventilation, days in the ICU, days in hospital, renal replacement therapy, and mortality were higher in ARDS due to COVID-19. However, in this study no increased risk of Right ventricular dysfunction was found and the presence of comorbidities did not increase the risk of morbidity/mortality.

### Electronic supplementary material

Below is the link to the electronic supplementary material.

Supplementary Material 1

Supplementary Material 2

## Data Availability

All data generated and analyzed during this study are included in this published article in its “Supplementary 1_Raw Data” file.

## Abbreviations

COVID-19: Coronavirus disease 2019
ARDS: Acute respiratory distress syndrome
ICU: Intensive care unit
PaO2/FiO2 ratio: Ratio of arterial oxygen partial pressure (PaO2 in mmHg) to fractional inspired oxygen
MV: Mechanical ventilation
ECMO: Extracorporeal Membrane Oxygenation
RV: Right Ventricle
COPD: Chronic Obstructive Pulmonary Disease
TAPSE: Tricuspid annular plane systolic displacement
PASP: Pulmonary artery systolic pressure
TAPSE/PSAP: Ratio of tricuspid annular plane systolic displacement to pulmonary artery systolic pressure
SAPS-II score: Simplified Acute Physiology Score II
APACHE-II score: Acute Physiology and Chronic Health Evaluation
SOFA score: Sequential Organ Failure Assessment score
RVD: Right ventricular dysfunction
FDA: Food and Drug Administration
NT-proBNP: N-terminal pro B-type natriuretic peptide
P-SILI: Pulmonary self induced lung injury
