# Supplementary material for: Acute respiratory distress syndrome in patients with COVID-19 vs. Non-COVID-19: clinical characteristics and outcomes in a tertiary care setting in Mexico City
Source: BMC Pulm Med. 2023 Nov 6;23:430. doi: 10.1186/s12890-023-02744-6 (PMC10626689; doi:10.1186/s12890-023-02744-6)
Supplement: Supplementary file 2 — Supplementary Material 2 [file 12890_2023_2744_MOESM2_ESM.docx]

| Appendix A. Comorbidities associated with Mortality: Multivariate Analysis | |
| --- | --- |
| Comorbidities | P value* (CI 95%) |
| Diabetes mellitus  Systemic Hypertension  COPD  Heart Failure  Ischemic Heart Disease | 0.18 (95% IC, 0.021-1.594)  0.83 (95% IC, 0.22-3.1)  0  0.93 (95% IC, 0.085-9.6)  0 |

*Chi square test was used.

Abbreviations: Chronic Obstructive Pulmonary Disease
